# Editorial: Recent progress on catalysis for energy and environmental applications

**DOI:** 10.3389/fchem.2025.1679169

**Published:** 2025-08-28

**Authors:** Weiwei Zhang, Tianwei He, Liang-Nian He, Yongjun Ji

**Affiliations:** ^1^ Beijing Technology and Business University, Beijing, China; ^2^ Yunnan University, Kunming, China; ^3^ Nankai University, Tianjin, China

**Keywords:** energy catalysis, environmental catalysis, industrial catalysis, synthetic strategy, catalytic performance

Energy sustainability and environmental protection are among the most pressing global challenges facing humanity. Rapid industrialization, accelerated urbanization, and continuous population growth have substantially exacerbated ecological issues, including air and water pollution, resource depletion, and climate change. Therefore, developing advanced catalytic technologies is critical to addressing these challenges and offers promising pathways toward renewable energy production, pollutant degradation, resource recovery, and emissions control (Zheng et al., 2022). Among various catalytic strategies, heterogeneous catalysis is particularly advantageous due to its high efficiency, ease of catalyst recovery, and broad applicability in industrial-scale processes (Zhu et al., 2024).

The Research Topic, “*Recent Progress on Catalysis for Energy and Environmental Applications*” highlights significant advancements in catalytic materials, innovative methodologies, and mechanistic insights pertinent to energy conversion and environmental purification. Comprising original research articles and comprehensive reviews, this Research Topic identifies contemporary scientific challenges and proposes novel catalytic approaches to enhance performance. It features four insightful contributions, each providing unique perspectives and practical implications across fields such as electrochemical sensing, catalytic oxidation, single-atom catalysts, and metal-organic frameworks (MOFs).

Yu et al. addressed the urgent need for rapid and sensitive detection of heavy metal ions in food and beverages by developing an innovative electrochemical sensor based on graphene (GR) combined with a covalent organic framework (COF). Contamination from heavy metals, such as Cd^2+^, Pb^2+^, and Cu^2+^, poses severe risks to human health and environmental safety. By synergistically integrating the superior electrical conductivity of GR with the high surface area and abundant active sites of the COF, the authors achieved the rapid and simultaneous detection of these toxic metals in Chinese liquor (Baijiu). The GR/COF-based electrochemical sensor demonstrated significantly enhanced sensitivity and practical application compared to traditional analytical methods, thereby facilitating efficient monitoring for food safety and environmental protection.

Catalytic oxidation of CO is essential for air purification and mitigating atmospheric pollution. To combat air pollution arising from industrial CO emissions, Luo et al. reported a novel Pt-Fe(OH)_*x*_ catalyst via a facile and efficient one-pot reduction method. The optimized Pt-Fe(OH)_*x*_ catalyst exhibited exceptional catalytic performance, achieving near-complete CO conversion at notably low temperatures (∼60 °C), and exhibiting excellent hydrothermal stability. Density Functional Theory (DFT) calculations emphasized the critical role of hydroxyl species at the Pt-Fe(OH)_*x*_ interface. These insights offer valuable guidance for designing robust and efficient catalysts suitable for practical industrial applications.

Liang et al. provided a comprehensive review of single-atom nanozymes (SAzymes) and their emerging applications in the biomedical and environmental fields. Despite their high catalytic efficiency, natural enzymes often suffer from environmental sensitivity, poor stability, and high production costs. SAzymes overcome these limitations through atomic-level dispersion and optimized catalytic efficiency, which is enabled by well-defined active sites. The review systematically summarized recent advances in SAzyme design, synthesis strategies, and catalytic mechanisms, emphasizing applications in oncology. Specifically, the authors demonstrated how SAzymes utilize substrates that are abundant in tumor microenvironments (such as H_2_O_2_) to generate reactive oxygen species for targeted cancer therapy. Additionally, key strategies to enhance SAzyme stability and specificity were discussed, underscoring their potential to revolutionize cancer treatment and environmental remediation by integrating biological specificity with inorganic robustness.

Finally, Zhang et al. addressed water pollution by improving the efficiency of Fenton-like reactions, a well-known advanced oxidation process (AOP) for degrading recalcitrant organic pollutants. The team developed amino-functionalized Fe/Co bimetallic MOF catalysts to facilitate redox cycling between Fe (III) and Fe (II), which is a rate-determining step in Fenton-like systems. The optimized NH_2_-MOF(Fe, Co) catalyst exhibited markedly enhanced degradation performance for sulfamethoxazole (SMX, a common pharmaceutical pollutant in wastewater). This dual-modification strategy, incorporating both NH_2_ functional groups and Co doping, improved electron transfer kinetics and redox reaction rates, resulting in significantly improved pollutant removal compared to conventional Fe-based MOFs. Comprehensive characterization techniques, including electron paramagnetic resonance and radical trapping experiments, clarified the degradation pathways and active species involved, offering valuable insight for designing effective wastewater treatment strategies under mild conditions.

Taken together, these contributions represent significant recent advancements in catalytic technologies for energy conversion and environmental remediation. They demonstrate how next-generation catalytic materials, innovative synthesis methods, and fundamental mechanistic understanding are essential for overcoming current technological limitations. This Research Topic expands on existing knowledge and provides practical guidance for real-world applications, thereby supporting global efforts toward environmental sustainability and a cleaner energy future.

As guest editors, we sincerely appreciate all the contributing authors for their excellent work and thank the reviewers for their constructive and insightful feedback. We anticipate that this Research Topic will stimulate continued innovation, foster cross-disciplinary collaborations, and facilitate the practical deployment of advanced catalytic technologies, ultimately contributing to a sustainable and healthier global environment.
